# Equestrian STAR: Development of an Experimental Methodology for Assessing the Biomechanical Performance of Equestrian Helmets

**DOI:** 10.1007/s10439-025-03723-0

**Published:** 2025-04-28

**Authors:** Lauren A. Duma, Mark T. Begonia, Barry Miller, Caitlyn Jung, Matthew Wood, Brock G. Duma, Steve Rowson

**Affiliations:** Virginia Tech Helmet Lab, Blacksburg, VA 24061 USA

**Keywords:** Equestrian, Helmet, Concussion, Rating, Injury, Fall

## Abstract

**Purpose:**

The current equestrian helmet standards set minimal requirements for passing helmets, highlighting the need for a rating system that differentiates helmets based on their impact performance. This study’s objectives were to compare equestrian helmet impact response kinematics between linear-driven and oblique impact conditions and then to evaluate the effect of incorporating oblique drop tests into a previously established equestrian helmet rating system, Equestrian STAR.

**Methods:**

Oblique drop tests were conducted with 45 equestrian helmet models at two impact locations, front boss and rear boss, at an impact velocity of 6.56 m/s. The resulting peak linear and rotational head accelerations were compared to those measured during linear-driven pendulum impacts on the same helmet models. A total of 720 impact tests were performed, making this the largest published study on equestrian helmets to date. Equestrian STAR was modified to include both pendulum and oblique impacts by computing and summing weighted concussion risks for each test condition.

**Results:**

Oblique impacts had peak linear accelerations ranging from 105.8 to 204.5 g and peak rotational accelerations ranging from 3304 to 13854 rad/s^2^. Between the linear-driven and oblique impacts, peak linear acceleration was weakly correlated (*R*^2^ = 0.34, *p* < 0.001), while peak rotational acceleration was not correlated (*R*^2^ = 0.04, *p* = 0.21). Equestrian STAR scores calculated using both pendulum and oblique impacts suggested that the worst-performing helmet on both systems had nearly four times the concussion risk as the best-performing.

**Conclusion:**

Pendulum and oblique impacts have different methods of generating head rotation, which can highlight different modes of helmet performance. The updated Equestrian STAR helmet rating system differentiates between high-performing and low-performing helmets, enabling equestrians to purchase helmets best at reducing concussion risk and providing companies with a process to compare their helmet designs.

## Introduction

Over 30 million people ride horses yearly in the USA, and there are over 50,000 emergency room visits due to equestrian injuries [1]. Equestrian sports encompass a wide range of disciplines, including Western riding, horse racing, and English events, such as dressage, show jumping, and cross country. Across these disciplines, the most common mechanism of injury is falling from the horse and impacting the ground [2, 3]. These falls result in equestrian sports having a higher risk of severe injury compared to both American football and motorcycling [4]. Moreover, equestrian sports are the greatest contributor of sports-related traumatic brain injuries (TBI), contributing 45% of all sports-related TBIs [5]. Of all injuries obtained through equestrian sports, head injuries are the most dangerous and life-threatening, resulting in the majority of hospitalizations and deaths from equestrian sports [4]. In addition to severe head injuries, equestrian falls frequently result in mild head injuries, such as concussions [6, 7]. Concussions are one of the most common injuries obtained in equestrian sports [8, 9].

When worn, helmets have been shown to reduce head injuries by 30% and severe head injuries by 50% in equestrian sports [10]. While this demonstrates that equestrian helmets can reduce head injury risk, the overall injury rates indicate that there is room for improvement in equestrian helmet design [11]. Given the high number of head injuries in equestrian sports, it is imperative to have high-quality helmets and intensive helmet standards to evaluate their impact performance. Currently, there are many pass/fail equestrian helmet standards used to regulate equestrian helmet performance (Table 1). These standards include the ASTM F1163-23, EN 1384:2023, NOCSAE ND050, PAS 015:2011, SNELL E2021, and VG1 01.040 [12–16]. Each of these standards involve linear drop tests onto flat, hazard, or hemisphere anvils. To pass the test, helmets must have a peak linear acceleration under a certain threshold. While these standards set minimum performance requirements for equestrian helmets, there are currently a lack of data available that can be used to compare equestrian helmets that have passed these standards.

**Table 1:** Current equestrian helmet standards arranged by impact velocity

Standard	Surface	Drop height (m)	Impact velocity (m/s)	Linear head acceleration limit	Rotational head acceleration limit
SNELL E2021	Flat anvil	1.87	6.06	275 g	None
ASTM F1163-23	Flat anvil	1.8	5.94	300 g	None
EN 1384:2023	Flat anvil	1.8	5.94	250 g	None
PAS 015:2011	Flat anvil	1.8	5.94	250 g	None
VG1 01.040	Flat anvil	1.8	5.94	250 g	None
NOCSAE ND050	Flat, hemisphere, and hazard anvils	1.5	5.46	1200 SI	None
SNELL E2021	Hemisphere anvil	1.5	5.42	275 g	None
SNELL E2021	Hazard anvil	1.31	5.07	275 g	None
ASTM F1163-23	Hazard anvil	1.3	5.05	300 g	None
EN 1384:2023	Hazard anvil	1.3	5.05	200 g	None
PAS 015:2011	Hazard anvil	1.3	5.05	200 g	None
NOCSAE ND050	Flat anvil	0.6	3.46	300 SI	None

As head impacts involve both linear and rotational acceleration, it is important to assess a helmet’s ability to reduce both. During a head impact, linear acceleration induces transient intracranial pressure gradients leading to focal injuries, while rotational acceleration increases brain strain, causing diffuse injuries [17–19]. Previous research has shown that rotational acceleration measurements are an important indicator of a helmet’s ability to reduce brain injury risk [18, 20–24]. To evaluate the role of rotational acceleration in head injuries specific to equestrian sports, studies by Bourdet et al. and Forero Rueda et al. used computer models to simulate head impacts observed in equestrian falls [3, 25]. These simulations demonstrated that rotational acceleration resulting from equestrian falls causes increased brain strain, and that angular kinematics should be included in equestrian helmet standards. Other research has also concluded that the current equestrian helmet standards, such as EN 1384, are not representative of real-world equestrian head impacts. Clark et al. found that the acceleration threshold for the EN 1384 standard was higher than reconstructed equestrian head impacts that resulted in concussions [26, 27]. These studies suggest that the current equestrian helmet standards do not thoroughly assess a helmet’s ability to reduce brain injuries.

To address the limitations of the injury criteria used by the current standards, Bourdet et al. developed a new comparative equestrian helmet testing methodology [28]. This system used the resultant linear and rotational accelerations from linear and oblique head impacts to estimate brain injury risk for 7 different equestrian helmet models. Folksam, a research group located in Sweden, created another equestrian helmet testing system for 13 different equestrian helmet models [29]. Their method involved a series of perpendicular and oblique impact tests that measured linear and rotational acceleration. Although both studies provide useful information on equestrian helmet performance, there are currently many more equestrian helmets available on the market.

The Summation of Tests for the Analysis of Risk (STAR) is another helmet rating system that has been developed for several sports, such as football, hockey, bicycling, soccer, snow sports, and whitewater [30–35]. The STAR helmet ratings are publicly accessible, published by Virginia Tech with the goal of supplementing current helmet standards and helping consumers compare the protective ability of the available helmets in their sport. Each STAR helmet rating system is based on observed head injury mechanisms for the specific sport, and lab testing involves multiple impact locations and velocities that match the sport’s individual injury mechanisms. Impact testing involves both centric and non-centric impacts, as previous studies have highlighted the importance of centric and non-centric configurations in concussion events [36–38]. The resultant peak linear and rotational head accelerations from impact tests are used to estimate concussion risks, which are multiplied by head impact exposure to calculate weighted injury risks for the given impact conditions. The weighted risks are summed to compute an overall STAR score, a single value that summarizes helmet performance across various sport-specific impact conditions. To aid in consumer interpretation, the STAR score is categorized into a rating out of 5 stars. A high rating indicates that a helmet is able to reduce head acceleration meaningfully and, therefore, also reduce concussion risk relative to other helmets in that sport. On-field studies have demonstrated that helmets highly rated by the STAR system are able to reduce head injury rates. For example, in a study of 1,833 football players across eight Division 1 universities, the 4-star Riddell Revolution reduced concussion risk by 54% compared to the 1-star Riddell VSR4 [39]. Further, helmets that reduce linear and rotational kinematics in the lab have been shown to reduce concussion risk on the field for NFL players [40].

A STAR rating system has been previously designed for equestrian helmets [41]. Equestrian STAR was developed through a series of studies to design a method of testing equestrian helmets in the lab that represents the head impacts equestrians experience in the field. Analysis of equestrian fall videos and previously published equestrian fall data were used to determine common head impact locations and fall heights (Appendix 1). Using these head impact configurations, equestrian helmets were tested on-field to generate linear and rotational acceleration traces of equestrian head impacts to the ground (Appendix 2). A pendulum impactor with a flat impactor surface, which was found to produce the highest head accelerations for the impact speeds tested (Appendix 3), was used for testing as it reasonably matched the acceleration magnitudes and durations measured on-field. Equestrian STAR tested 49 equestrian helmet models on the pendulum impactor, finding a wide range in equestrian helmet performance under linear-driven impact conditions. The best-performing helmet had a STAR score 88% lower than the worst-performing helmet, representing a significant reduction in predicted concussion rates.

Recently, we have considered incorporating oblique testing into Equestrian STAR. The previous version of Equestrian STAR does not consider high-speed falls where the head impacts the ground at an angle, which have been observed in horse racing and hunter/jumper events [42]. Falls in these events frequently occur while the horse is galloping, resulting in oblique head impacts with both normal and tangential velocity components. Further, the International Federation for Equestrian Sports (FEI) has proposed a new standard to assess equestrian helmet performance under oblique impact conditions. The FEI recommended that equestrian helmets be tested on an oblique drop tower at an impact velocity of 6.56 m/s and that passing helmets have a peak linear acceleration under 150 g and a peak rotational acceleration under 5500 rad/s^2^. These acceleration thresholds represent approximately a 50% risk of concussion, estimated through the Virginia Tech concussion risk function (Eq. 4). The 6.56-m/s impact velocity corresponds to a 2.2 m drop height, which matches the average height of an equestrian’s head while on a horse and is in the upper range of fall heights possible in equestrian sports.

This study evaluated equestrian helmet performance under oblique impact conditions and compared the oblique impacts recommended by the FEI to the linear-driven impacts previously performed in Equestrian STAR. If oblique testing provides different information about equestrian helmet performance than pendulum testing, then Equestrian STAR will be updated to include data from oblique impacts. The updated version of Equestrian STAR will address the limitations in the current equestrian helmet standards by including rotational kinematics, both linear-driven and oblique impacts, concussion risk assessment, and equestrian fall exposure.

## Methods

We selected a wide range of equestrian helmet models to use in oblique testing according to recommendations from the FEI. Helmet performance under oblique impact conditions was compared to linear-driven helmet tests performed in the previous Equestrian STAR testing protocol, specifically focusing on the correlation between peak linear and rotational acceleration within and between helmet testing systems. This analysis informed the decision to incorporate oblique testing into an updated Equestrian STAR rating system, and the subsequent impact on the Equestrian STAR helmet ratings was examined.

### Helmet Models

A selection of 45 popular equestrian helmet models available on the market were chosen for oblique testing (Table 2). These 45 helmet models were previously evaluated in the original Equestrian STAR helmet rating system and had passed at least one of the current equestrian helmet standards. Helmet sizes were chosen based on the circumference of the headform used in testing, and the helmet price was recorded upon purchase. Many helmets, 15 of the 45, implemented Multi-directional Impact Protection System (MIPS) technology. MIPS is a helmet insert developed to reduce rotational motion by adding a low-friction layer between the helmet liner and the wearer’s head. The presence or absence of MIPS was also recorded for each helmet.

**Table 2:** Equestrian helmet models selected for Equestrian STAR testing

Brand	Model	Abbreviation	Price ($)	MIPS	Size
Champion	Revolve Pro Plus MIPS Jockey	CRPPJM	187	Yes	58 cm
Champion	Revolve Vent-Air MIPS Jockey	CRVJM	299	Yes	58 cm
Champion	Revolve Vent-Air MIPS Peaked	CRVPM	412	Yes	58 cm
Champion	Revolve X-Air MIPS	CRXAM	460	Yes	58 cm
Champion	Revolve X-Air MIPS Peaked	CRXAPM	239	Yes	58 cm
Champion	X-Air Plus Jockey	CXAP	179	No	58 cm
Charles Owen	4Star	Co4S	375	No	58 cm
Charles Owen	AYR8 Plus	CoA8P	549	No	58 cm
Charles Owen	EQX Kylo	CoEK	185	Yes	Medium
Charles Owen	Halo MIPS	CoHM	549	Yes	58 cm
Charles Owen	MS1 Pro MIPS	CoMS1PM	249	Yes	58 cm
Charles Owen	My PS MIPS	CoMPSM	319	Yes	58 cm
GPA	Speed Air 2X	GSA2X	669	No	58 cm
IRH	4G	I4G	230	No	Large
IRH	Equi-Lite	IEL	50	No	Medium
IRH	Equi-Pro II	IEP2	60	No	Medium/large
IRH	Equi-Pro SV	IEPSV	70	No	Medium/large
IRH	Medalist	IM	100	No	7 1/8
Kask	Dogma	KD	599	No	58 cm
Kask	Kooki	KK	449	No	58 cm
Kask	Kooki Lady	KKL	449	No	58 cm
Kask	Star Lady	KSL	640	No	58 cm
KEP	Cromo 2.0	KeC2	649	No	58 cm
One K	Avance CCS MIPS Wide Brim	OkACCSM	370	Yes	Large
One K	Defender	OkD	280	No	Large
One K	Defender 2023	OkD23	290	No	Large
Ovation	Deluxe Schooler	ODS	70	No	Medium/large
Ovation	Jump Air	OJA	130	No	Medium/large
Ovation	Sync	OS	85	No	Medium/large
Resistol	Straw Ridesafe	RSRS	185	No	Large
Samshield	Shadowmatt	SS	549	No	58 cm
Tipperary	Sportage hybrid	TiSH	150	No	XL
Tipperary	Windsor MIPS	TiWM	400	Yes	Large
Trauma Void	EQ3 MIPS	TvEQ3M	249	Yes	58 cm
Trauma Void	Lynx Eventing MIPS	TvLEM	249	Yes	58 cm
Trauma Void	Lynx MIPS	TvLM	249	Yes	Medium
Trauma Void	Pardus MIPS	TvPM	289	Yes	Medium
Troxel	ES	TrES	145	No	Medium
Troxel	Spirit	TrS	69	No	Medium
Troxel	Spirit low profile	TrSLP	75	No	Medium
Troxel	Sport 2.0	TrS2	46	No	Medium
Troxel	Terrain	TrT	120	No	Medium
TuffRider	Carbon Fiber	TfCFS	58	No	Medium
TuffRider	Ventek	TfV	81	No	7 1/8
Uof	Race	UofR	357	No	58 cm

### Oblique Impact Testing

Oblique testing was performed on an oblique drop tower. The oblique drop tower drops a supported helmeted 50th percentile male NOCSAE headform (Southern Impact Research Center, Rockford, TN) without a neck onto a 45° steel anvil covered in 80-grit sandpaper. The NOCSAE headform has a circumference of 57.6 cm and its shape provides a realistic fit with the helmet [43]. To ensure proper fit between the helmet and headform, the helmet rim is positioned 2.5 cm above the brow line, the straps are secured tightly under the chin, and the adjustment dials are tightened until resistance is met. The helmeted headform is held in place by a support ring and is positioned by adjustable rods inside the ring. An inclinometer (WT9011DCL, WitMotion Shenzhen Co., Ltd, ShenZhen, China) is used to assess correct x and y rotation, and consistent z rotation is verified with a cross-level laser and 5° increments inscribed on the support ring. The support ring is attached to the drop tower and a lever arm, which releases the headform before it impacts the anvil. During the impact, the support ring passes around the angled anvil. The oblique drop tower generates impacts with relative normal and tangential velocity vectors.

To measure impact response kinematics, the NOCSAE headform was instrumented with a six-degree-of-freedom sensor package: three linear accelerometers (Endevco 7264B-2000, PCB Piezotronics, Depew, NY) and a tri-axis angular rate sensor (DTS ARS3 PRO, Diversified Technical Systems Inc., Seal Beach, CA) at the headform’s center of gravity. Data were sampled at 20 kHz and filtered using a 4-pole Butterworth low pass filter. Linear acceleration data were filtered with a cut-off frequency of 1650 Hz (CFC 1000) according to SAE J211 and angular rate data were filtered with a cut-off frequency of 300 Hz (CFC 180). Rotational accelerations were calculated through five-point stencil differentiation of the angular rate data [44].

**Fig. 1:**
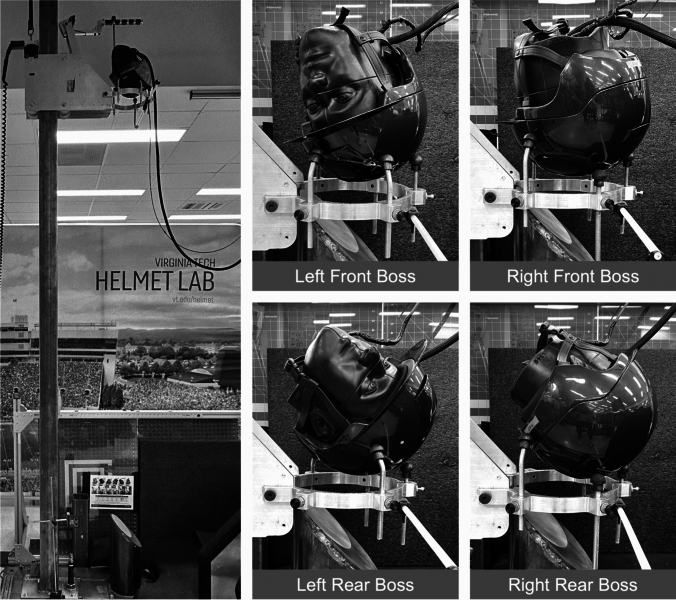
The oblique drop tower and four impact locations used in oblique testing (front boss is the average of left and right front boss, rear boss is the average of left and right rear boss)

Four locations were assessed on each helmet model: the left front boss, right front boss, left rear boss, and right rear boss (Fig. 1, Table 3). As each location was only impacted once, one helmet was purchased and tested for each helmet model. Data from the left and right front boss were averaged into one value for the front boss, and data from the left and right rear boss were averaged into one value for the rear boss. These locations were chosen to complement the three locations previously tested in Equestrian STAR (the front, side, and back) to provide a more comprehensive evaluation of head acceleration reduction across the helmet. Both the front boss and rear boss locations were impacted at 6.56 m/s. Testing the 45 equestrian helmet models under these conditions resulted in a total of 180 impact tests.

**Table 3:** NOCSAE headform rotations on the support ring for each oblique impact location

Location		X (deg)	Y (deg)	Z (deg)
Front boss	Left	15.6	5.7	− 80
	Right	− 15.6	5.7	80
Rear boss	Left	46.0	− 27.9	170
	Right	− 46.0	− 27.9	− 170

X and Y rotations were determined with an inclinometer according to the SAE J211 coordinate system, and Z axis rotation was determined by projecting the midsagittal line (for front boss locations) or mid-coronal line (for rear boss locations) of the headform face onto the support ring marked with 5° increments. A 0° position corresponds to the headform facing the drop tower, with positive Z values increasing in the clockwise direction and negative in the counterclockwise direction

### Pendulum Impact Testing

The oblique tests were compared to the linear-driven impact tests previously performed in Equestrian STAR testing. Helmets were tested on a pendulum impactor, which uses a pendulum arm connected to an impactor surface to induce linear-driven impacts toward the headform’s center of gravity. Equestrian STAR testing used a flat impactor surface that was 12.7 cm wide, 2.5 cm thick, and composed of nylon. The pendulum impactor uses the same NOCSAE headform and instrumentation described for the oblique drop tower. The headform is mounted to a Hybrid III neck with a custom adaptor plate, which is designed to give the headform anatomically accurate locations of the headform center of gravity and occipital condyle pin [45]. Among the available options, the Hybrid III neck is the most commonly used in helmet testing, including NOCSAE and NFL testing. Additionally, peak kinematics occur prior to any appreciable neck flexion or hyperextension. The headform and neck are secured to a 16 kg sliding mass and adjustable table (Biokinetics, Ottawa, Ontario, Canada), which mimics a 50th percentile male’s effective torso mass during a head impact. The pendulum arm is composed of 10.16 × 5.8 cm rectangular aluminum tubing, is 190.5 cm long, and has a moment of inertia of 72 kg m^2^. The arm has a total mass of 36.3 kg, including the 16.3-kg impacting mass. The pendulum arm is raised and released by a winch system and electromagnet.

**Fig. 2:**
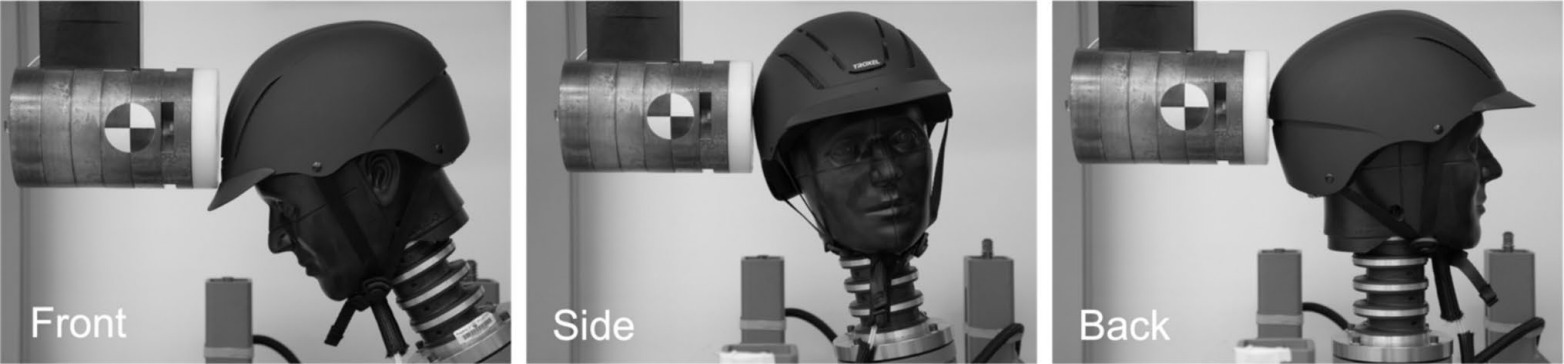
The front, side, and back impact locations used in Equestrian STAR pendulum impactor testing

The pendulum impactor was used to impact three locations: the front, side, and back (Fig. 2, Table 4). The three locations were tested at two impact speeds, 4.0 m/s and 6.3 m/s, for a total of six impact conditions. The 4.0-m/s and 6.3-m/s impact speeds were chosen to represent the range of fall heights present in equestrian sports (Appendix 1) [46]. For each helmet model, the six impact conditions were tested twice and averaged. The 45 equestrian helmet models tested in these six impact configurations resulted in a total of 540 impact tests. Four helmets of each model were purchased, for a total of 180 helmets, to ensure that each impact location was only impacted once per helmet. Each of the four purchased helmets were impacted 3 times, each impact in a different location to prevent any deformation from influencing subsequent impact tests.

**Table 4:** NOCSAE headform translations and rotations on the linear slide table using the SAE J211 coordinate system for each pendulum impact location

Location	Y (cm)	Z (cm)	Ry (°)	Rz (°)
Front	0	+ 0.8	− 30	0
Side	− 1	+ 4.8	− 10°	− 100
Back	0	+ 6.3	0°	180

The zero position was defined as 0° rotation about the Y and Z axes, with the intersection of the headform’s midsagittal and transverse planes aligned to the center of the impactor

### Statistical Analysis

To compare the oblique drop tower and pendulum impactor’s resultant linear and rotational head accelerations, we calculated the Pearson’s product-moment correlation coefficients between the peak accelerations within each system and across the two systems (R version 4.4.1, RStudio; Boston, Massachusetts, USA). For each of the helmet models, we first averaged the peak linear and rotational accelerations across impact conditions (location and speed) within each system. By averaging across impact conditions, we examined the relationship between the peak accelerations generated on each test system by helmet model. We then computed the correlation between peak linear and rotational accelerations within the oblique drop tower, peak linear and rotational accelerations within the pendulum impactor, peak linear accelerations across the two systems, and peak rotational accelerations across the two systems.

### Equestrian STAR Helmet Rating System

The results from the statistical analysis suggested that unique information on equestrian helmet performance is provided through oblique testing. Therefore, we decided to incorporate oblique tests into the Equestrian STAR helmet rating system as recommended.

The Equestrian STAR score was calculated using the resultant linear and rotational head accelerations from pendulum and oblique impact testing (Eq. 1). The Equestrian STAR equation summarizes data from 8 impact conditions into one overall score: 6 impact conditions on the pendulum impactor (the front, side, and back at 6.3 m/s and 4.0 m/s) and 2 impact conditions on the oblique drop tower (the front boss and rear boss at 6.56 m/s). The overall Equestrian STAR equation is broken into two equations, one for pendulum impactor testing and a second for oblique drop tower testing (Eqs. 2 and 3). By calculating the STAR scores for pendulum and oblique impacts separately and then combining them, the performance quantified by the impacts in both systems are weighted equally. In both equations, *E* represents exposure, which is a function of location (*L*) and impact velocity (*V*). *R* represents concussion risk, which is a function of peak linear acceleration (*a*) and peak rotational acceleration (*∝*).

1 $$Equestrian STAR=E{questrian STAR}_{pendulum}+{Equestrian STAR}_{oblique}$$

2 $${Equestrian STAR}_{pendulum}=\sum_{L=1}^{3}\sum_{V=1}^{2}E\left(L,V\right)*R\left(a,\propto \right)$$

3 $${Equestrian STAR}_{oblique}=\sum_{L=1}^{2}\sum_{V=1}^{1}E\left(L,V\right)*R\left(a,\propto \right)$$

Exposure values for each impact condition were assigned based on equestrian fall data. In our previous analysis of equestrian fall videos, we found that the front, side, and back of the head are all commonly impacted (Appendix 1). This is supported by other published studies, which also found that locations across equestrian helmets are impacted in similar frequencies during equestrian falls [46, 47]. Given that each head location is commonly impacted in equestrian environments, each impact location was treated as having a similar exposure (Table 5).

**Table 5:** Exposure values for each head impact condition (n/a = not applicable)

Impact location	Pendulum		Oblique
	4.0 m/s	6.3 m/s	6.56 m/s
Front	3	1	n/a
Side	3	1	n/a
Back	3	1	n/a
Front boss	n/a	n/a	1
Rear boss	n/a	n/a	1

Through our video analysis, we found that most equestrian falls occur from lower, reduced fall heights, which are approximately 3 times as frequent as falls from the full height of the horse (Appendix 1). Similar to the results from our video analysis, a study by Connor et al. also found that most equestrian falls occur at lower impact speeds, between 3.5 m/s and 4.0 m/s [46]. Based on data from our video analysis and previous studies, the low-energy tests were assigned a higher weighting than the high-energy tests, given that riders experience the low-energy impacts more often. The low-speed impacts at 4.0 m/s were assigned an exposure value of 3, and the high-speed impacts were assigned a value of 1 (Table 5).

The bivariate injury risk function, *R* ($a,\propto )$, calculates the probability of a concussion resulting from an impact for a given peak linear and rotational head acceleration (Eq. 4). The risk function was developed using head acceleration data collected from high school and college football players [18, 24]. Using a multivariate logistic regression analysis, concussion risk was modeled as a function of both linear and rotational head acceleration. The ability of the risk function to accurately predict concussion risk was assessed through NFL head impact reconstructions along with the original impact data used to create the risk function.

4 $$R(a,\propto ) =\frac{1}{1 + {e }^{- ( -10.2 + 0.0433 * a + 0.000873 * \propto - 0.000000920 * a\propto )}}.$$

For each of the eight impact conditions, the calculated risk is multiplied by the exposure for the tested impact location and speed to compute a weighted risk of concussion. These eight weighted risks are summed into the overall STAR score, which is then used to determine a helmet model’s star rating for consumer interpretation. Star ratings range from 0 to 5 stars, with 5-star ratings given to helmets that greatly reduce concussion risk. Because lower STAR scores indicate a lower risk of concussion, helmets with a low STAR score will have ratings with more stars.

The star rating thresholds were set after calculating the STAR scores for the 45 selected equestrian helmets. Each threshold was set relative to the mean STAR score: the 5-star threshold represented a 50% reduction in STAR score compared to the mean, and each subsequent threshold was established by incrementally increasing the required STAR score by 50% of the 5-star threshold.

## Results

### Oblique and Linear-Driven Impact Comparison

**Fig. 3:**
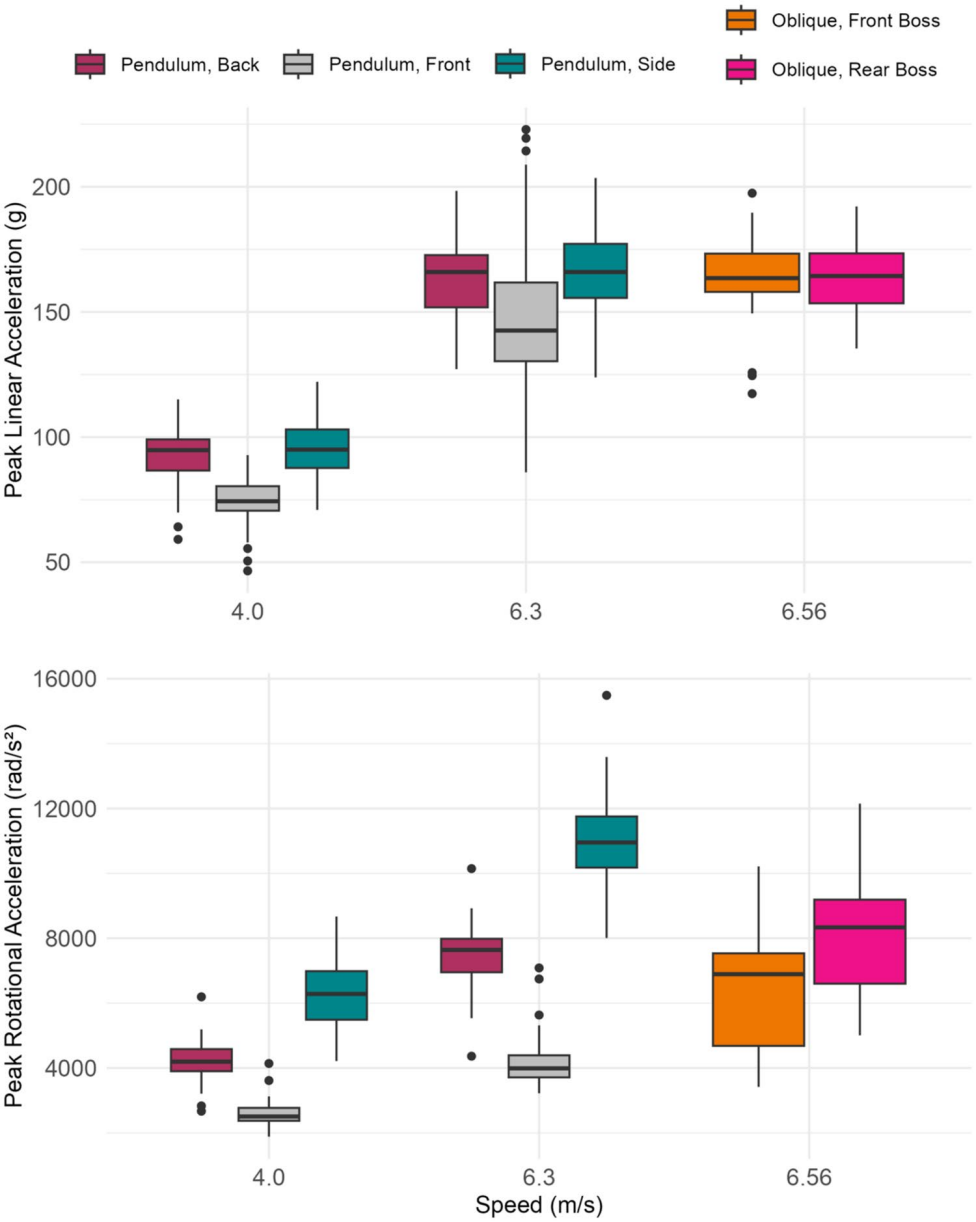
Peak linear and rotational acceleration distributions for the 45 equestrian helmet models tested in 8 impact configurations: the back, front, and side at 4.0 m/s and 6.3 m/s on the pendulum impactor, and the front boss and rear boss at 6.56 m/s on the oblique drop tower

For each of the eight impact conditions, the 45 helmet models had large ranges in peak linear and rotational acceleration (Fig. 3). For linear-driven impacts on the pendulum impactor, peak linear acceleration ranged from 43.9 to 126.4 g at 4.0 m/s and 86.0 to 230.2 g at 6.3 m/s. Peak rotational acceleration ranged from 1883 to 8694 rad/s^2^ at 4.0 m/s and 3128 to 15513 rad/s^2^ at 6.3 m/s. Oblique impacts at 6.56 m/s on the oblique drop had peak linear accelerations ranging from 105.8 to 204.5 g and peak rotational accelerations ranging from 3304 to 13854 rad/s^2^. Average peak linear and rotational accelerations for each helmet model are included in Appendix 4.

**Fig. 4:**
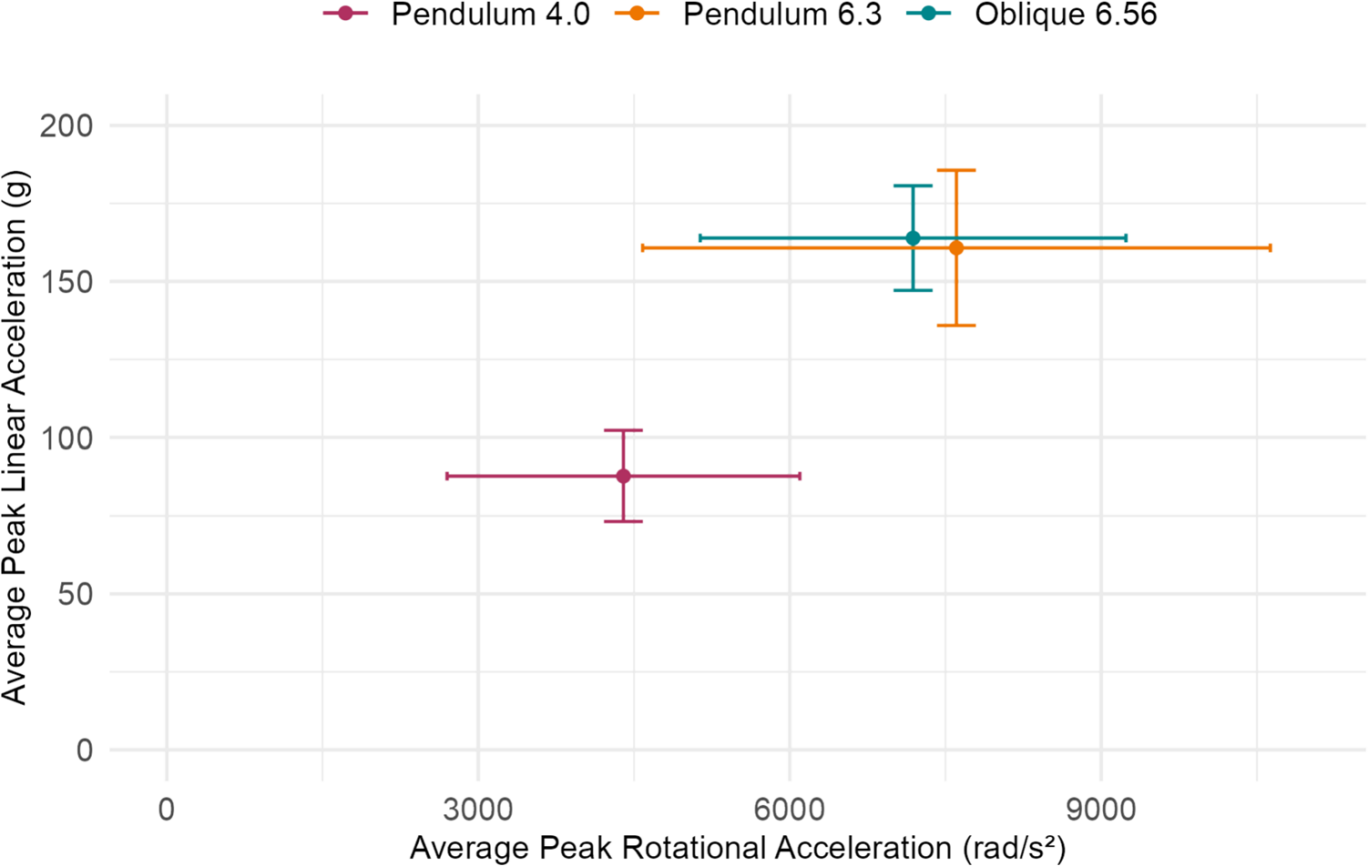
Average peak linear and rotational acceleration across the 45 equestrian helmet models tested on the pendulum impactor at 4.0 m/s and 6.3 m/s and on the oblique drop tower at 6.56 m/s. The error bars represent one standard deviation above and below the mean

High-speed impacts on the pendulum impactor and oblique drop tower had similar average peak linear and rotational accelerations. Across the 45 tested equestrian helmet models, oblique impacts at 6.56 m/s had an average peak linear acceleration of 163.9 g and an average peak rotational acceleration of 7187 rad/s^2^ (Fig. 4). These impacts were similar in severity to linear-driven pendulum impacts at 6.3 m/s, which had an average peak linear acceleration of 160.7 g and an average peak rotational acceleration of 7606 rad/s^2^. Pendulum impacts at 4.0 m/s were lower in severity, with an average peak linear acceleration of 87.7 g and an average peak rotational acceleration of 4398 rad/s^2^.

**Fig. 5:**
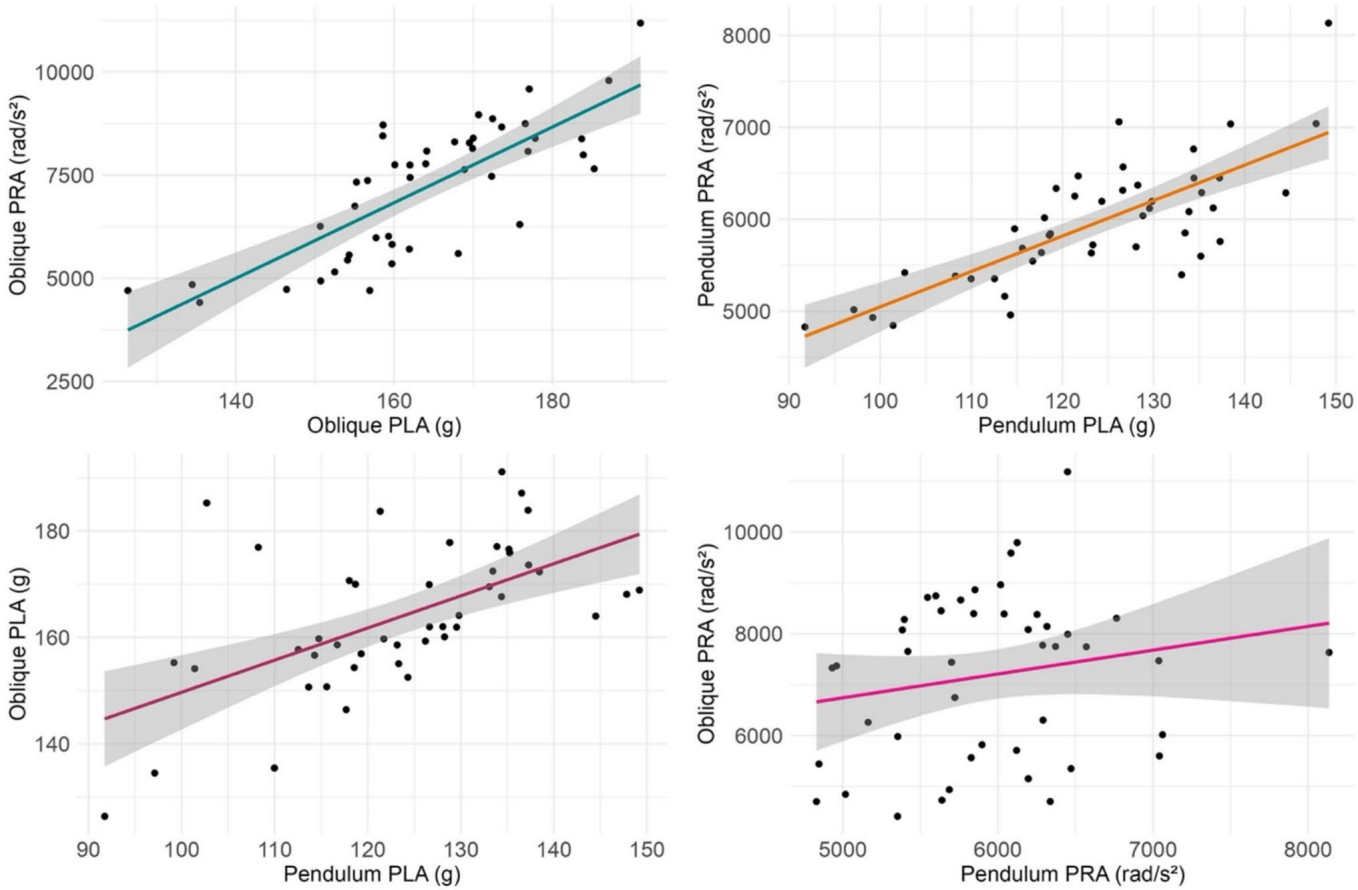
Peak linear acceleration (PLA) and peak rotational acceleration (PRA) within systems (top) and between the oblique drop tower and pendulum impactor (bottom) for the 45 tested equestrian helmet models, showing a moderate correlation between PLA and PRA within systems, a weak correlation between PLA in oblique and pendulum impacts, and no correlation between PRA in oblique and pendulum impacts

Although the pendulum impactor and oblique drop tower produced impacts that were similar in overall severity, the peak linear and rotational acceleration correlations between the two systems suggested that they had different methods of generating head rotation (Fig. 5). Within systems, peak linear and rotational acceleration were similarly correlated. Peak linear and rotational acceleration were moderately correlated across impacts on the oblique drop tower (*R*^2^ = 0.60, *p* < 0.001) and were also moderately correlated across impacts on the pendulum impactor (*R*^2^ = 0.59, *p* < 0.001). Between the pendulum impactor and oblique drop tower, peak linear acceleration was weakly correlated (*R*^2^ = 0.34, *p* < 0.001), showing that helmets with lower linear accelerations in the pendulum impacts generally also had lower linear accelerations in the oblique impacts. However, we did not observe this same trend with rotational acceleration. Peak rotational acceleration was not correlated (*R*^2^ = 0.04, *p* = 0.21) between the two test systems, suggesting that relative helmet performance varies between the two systems.

### Equestrian STAR Helmet Rating System

Incorporating oblique testing into the Equestrian STAR equation resulted in a wide range of Equestrian STAR scores across the 45 helmets, highlighting the performance differences between each model. STAR scores ranged from 2.11 to 8.03, with an average STAR score of 4.90. The best-performing helmet had a STAR score 73.7% lower than the worst-performing helmet, demonstrating a substantial reduction in concussion risk. On average, the pendulum component of the STAR score composed 67.5% of the overall STAR score. As there were fewer oblique impact conditions, the oblique tests composed 32.5% of the average overall STAR score. The pendulum components, oblique components, and total STAR scores for each helmet model are included in Appendix 4.

An independent t-test revealed that helmets with MIPS had significantly lower STAR scores (*M* = 4.04, SD = 1.14) compared to helmets without MIPS (*M* = 5.05, SD = 1.02) (*p* < 0.001). Helmets with MIPS generally performed better throughout testing, with lower peak accelerations compared to helmets without MIPS. Interestingly, helmet price was not correlated to the STAR score (*R*^2^ = 0.02, *p* = 0.34), showing that some expensive helmets performed poorly while some inexpensive helmets performed very well.

**Fig. 6:**
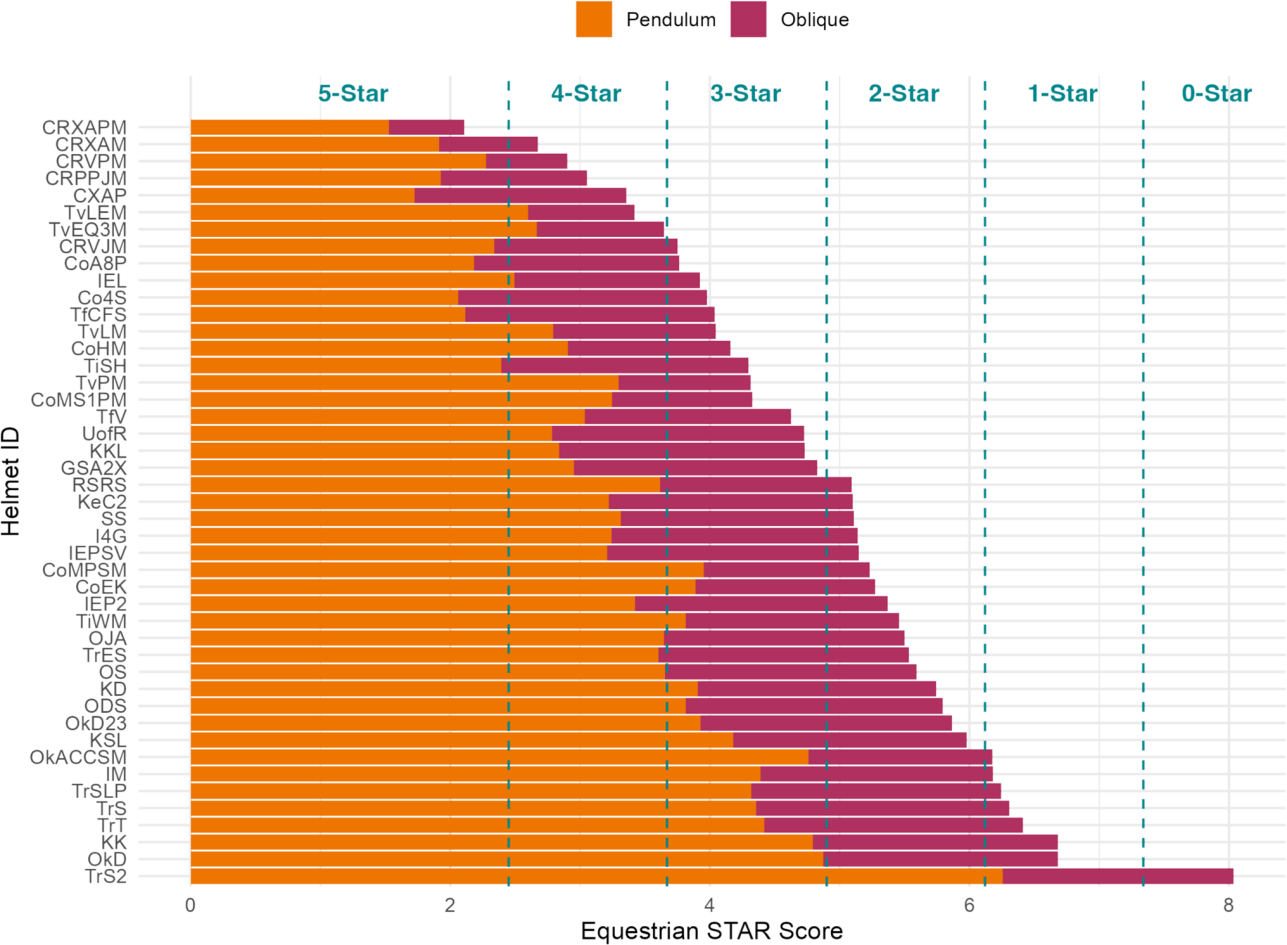
Equestrian STAR scores and ratings for the 45 tested equestrian helmet models, showing the portions of the STAR score contributed by pendulum and oblique testing

The average STAR score, 4.90, was used to set the star rating thresholds as described in the methods (Table 6). The 5-star threshold was set to 2.45, a 50% reduction in the mean STAR score. The 4-star threshold was then set to 3.67 (1.5 times the 5-star threshold), the 3-star threshold set to 4.90 (2 times the 5-star threshold), the 2-star threshold set to 6.12 (2.5 times the 5-star threshold), and the 1-star threshold set to 7.34 (3 times the 5-star threshold). With these thresholds, 1 helmet model received a 5-star rating, 6 received a 4-star rating, 14 received a 3-star rating, 16 received a 2-star rating, 7 received a 1-star rating, and 1 received 0 stars (Fig. 6).

**Table 6:** Equestrian STAR rating thresholds

STAR score	Star rating
< 2.45	5
< 3.67	4
< 4.90	3
< 6.12	2
< 7.34	1
≥ 7.34	0

## Discussion

This study compared equestrian helmet performance between linear-driven and oblique impact conditions and provided support for incorporating oblique testing into an updated version of the Equestrian STAR helmet rating system. Rotational acceleration was not correlated between oblique impacts on the oblique drop tower and linear-driven impacts on pendulum impactor, suggesting that oblique testing provides additional information on equestrian helmet performance. By integrating a tangential velocity vector to the impact direction, the oblique drop tower has a distinct method of inducing head rotation that is present in high-speed falls in specific equestrian events such as horse racing. As both linear-driven and oblique head impacts are possible in equestrian environments, including both in Equestrian STAR enables a more comprehensive evaluation of equestrian helmet performance.

The Equestrian STAR testing methodology was able to identify a large range in equestrian helmet performance, with STAR scores ranging from 2.11 to 8.03. Other STAR rating systems, such as hockey, bicycle, and whitewater, similarly demonstrated large performance differences between helmets within their sport [31, 32, 35]. For example, Whitewater STAR also had a large range in STAR scores from 0.25 to 4.86. However, in comparison to Football STAR, Equestrian STAR had far fewer highly rated helmets. Equestrian STAR only has one 5-star rated helmet, while Football STAR currently has 31 5-star rated helmets. Interestingly, when Football STAR ratings were first released in 2011, there was also only one 5-star rated helmet. Looking forward, equestrian helmets could be improved by incorporating the changes in helmet design seen in other sports over the past decade.

The updated Equestrian STAR rating system will help equestrian riders understand how the available equestrian helmets compare to each other in their ability to reduce both linear and rotational head acceleration. The current standards for equestrian helmets are pass/fail only, offering no distinctions between the most and least protective helmets. Because equestrian helmet price is not correlated to performance, price also fails to distinguish between high- and low-performing helmets. Further, while helmets with MIPS had significantly lower STAR scores, their performance varied widely, with ratings ranging from 1 to 5 stars. This variability in performance can be attributed to other differences in helmet design, such as padding type, thickness, or how MIPS was incorporated into the helmet. By giving equestrians the information needed to purchase the most protective helmets, the severity of head injuries caused by equestrian falls has the potential to decrease. Equestrian STAR can also help equestrian helmet companies design safer helmets by providing them with a process to compare the effectiveness of their helmet models.

In addition to providing comparable information on helmet performance, Equestrian STAR involves more rigorous impact testing than the available equestrian helmet standards: ASTM F1163-23, EN 1384:2023, NOCSAE ND050, PAS 015:2011, SNELL E2021, and VG1 01.040 (Table 1). The high-energy impact velocities tested in Equestrian STAR, 6.3 m/s and 6.56 m/s, are higher than all of the impact velocities tested in the current standards, which range from 3.46 m/s to 6.06 m/s. Equestrian STAR also encourages larger reductions in head acceleration, with helmets needing to minimize both linear and rotational acceleration to achieve a high rating. This is unique from the standards, which only implement relatively high linear acceleration thresholds ranging from 200 g to 300 g. Finally, Equestrian STAR requires helmets to perform well in both linear-driven and oblique impacts to achieve a high rating, while the standards only involve linear drop tests. Because the current standards have minimal requirements, they are unable to incentivize advances in equestrian helmet design. Equestrian STAR will encourage helmet manufacturers to improve their helmet designs to achieve higher ratings and allow them to sell safety through marketing higher ratings. While the equestrian helmet standards have several limitations, they are still useful to consider alongside the Equestrian STAR ratings when selecting an equestrian helmet. The Equestrian STAR ratings have no set pass/fail requirements and are instead designed to supplement the available standards by providing information on equestrian helmet performance relative to other equestrian helmets.

Previous equestrian helmet evaluation systems have also found performance differences between helmet models. For example, in the evaluation system developed by Bourdet et al., the best-performing helmet had a 43% mean brain injury risk, compared to 60% for the worst-performing helmet [28]. Consistent with Equestrian STAR, the rating system by Bourdet et al. found that Champion helmets were among the best-performing. Another evaluation system by Folksam also identified large performance differences between helmets, with the best helmet performing 40% better than the average helmet and the worst helmet performing 11% worse [29]. Few helmets overlapped between the Folksam evaluation system and Equestrian STAR, making a direct comparison of the results difficult. However, the helmets rated by both Folksam and Equestrian STAR had slightly different rank orders, which could be explained by differences in testing methodologies. Folksam did not evaluate helmets in low-speed impacts, tested fewer locations across the helmet, and gave oblique impacts a higher weighting. Although different, the information provided by Folksam on brain strain through finite element modeling is useful to consider alongside Equestrian STAR when purchasing a helmet.

Along with updating Equestrian STAR to include oblique impacts, we performed oblique testing to address the new equestrian helmet acceleration thresholds proposed by the FEI: a peak linear acceleration under 150 g and a peak rotational acceleration under 5500 rad/s^2^ during oblique impacts at 6.56 m/s. Of the 45 equestrian helmet models tested under these conditions, only 4 passed the FEI thresholds at both the front boss and rear boss locations. Although few helmets were able to pass this standard, we do not suggest that this was a poor recommendation. Instead, so few helmets passing the FEI standards further emphasizes the current shortcomings in equestrian helmet design, and its implementation can help advance equestrian helmet performance by serving as a goal for helmet manufacturers. It is important to note, however, that we tested the exact specifications recommended by the FEI with the exception of headform type. While different headforms might produce slightly different results, we would expect generally similar findings [48, 49].

Translating on-field equestrian head impacts into a laboratory environment resulted in limitations in our testing methodology. First, the frictional interfaces of the flat impactor used in pendulum testing and the sandpaper-covered anvil used in oblique testing did not necessarily represent those of the sand, dirt, and grass surfaces found in equestrian sports. Second, although equestrian helmets are marketed for single-use, multiple locations were impacted on each helmet during pendulum and oblique testing. However, the same location was never impacted more than once on the same helmet, and there was no visible deformation to any impact location prior to testing. Single-use helmet testing protocols set by the Consumer Product Safety Commission (CPSC) suggest that separating impact locations by more than 120 mm is sufficient to prevent any interactions between impact tests performed on the same helmet [50]. Throughout Equestrian STAR testing, all impact locations were set greater than 120 mm apart. While we tested helmets according to single-use guidelines, it is interesting to note that a recent study found 78% of riders do not replace their helmets after fall, despite the helmets being marketed as single use [51]. Next, while equestrian sports have a large female population, the concussion risk function used in Equestrian STAR was developed using football players, who are predominantly male. This may lead to an underestimation of concussion risk, as studies indicate that female athletes have higher concussion injury rates in sex-comparable sports [52]. However, in populations including both males and females, such as in automotive crashes, the concussion risk function has proven to be a better predictor of concussion than other injury metrics [53]. Lastly, a future direction to this study could investigate the influence of helmet design on the attenuation or aggravation of cervical spine injuries, which can be severe in equestrian falls. Despite these limitations, Equestrian STAR is still able to provide valuable information on differences in equestrian helmet performance relative to head acceleration reduction.

An additional limitation to this study was in our statistical analysis of the oblique drop tower and pendulum impactor. During Equestrian STAR testing, different impact locations and energies were tested on the oblique drop tower than the pendulum impactor. The resulting peak accelerations from these impacts were then used to calculate the correlation between the peak linear and rotational accelerations generated by the two systems. If we matched the impact energies and locations across the oblique drop tower and pendulum impactor, we suspect that the correlation in linear acceleration between the two systems would increase, but the same would likely not be true for rotational acceleration.

Overall, the results from this study emphasize the need for improvement in many equestrian helmet designs. This could be accomplished through a combination of experimental and computational methods. Equestrian helmets can also be tested by replicating falls with a sled-mounted dummy, which allows for the influence of the body on head impact kinematics to be examined [54]. Other studies have used acceleration data from impact tests to calculate brain strain-based injury criteria for helmet evaluation [55–58]. In addition to brain injury analysis, computer modeling has many applications in helmet design. Finite element models have been used to optimize helmet prototypes by assessing the effectiveness of their components, such as the helmet shell and padding [59–62]. These techniques, along with the Equestrian STAR evaluation system, could be used to design equestrian helmets that better reduce the risk of brain injuries. Ultimately, head injury rates in equestrian sports can be lowered by encouraging riders to wear helmets better at lowering head accelerations.

## Figures and Tables

**Fig. 7:**
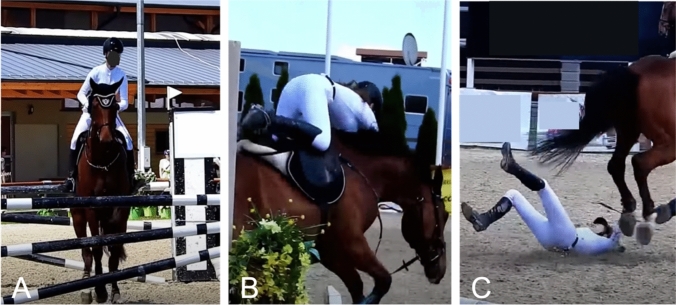
For each fall event, a picture of the beginning (**A**), the actual drop height (**B**), and the impact location (**C**) were analyzed. Images from Ján Yanco Čepa

**Fig. 8:**
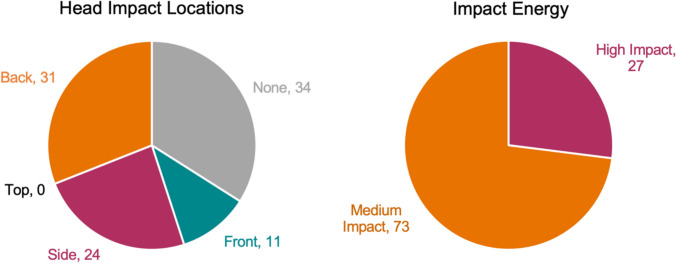
The location and number of head impacts observed in the 100 equestrian falls and the percentage of the falls involving head impacts that had a high fall height or a medium fall height

**Fig. 9:**
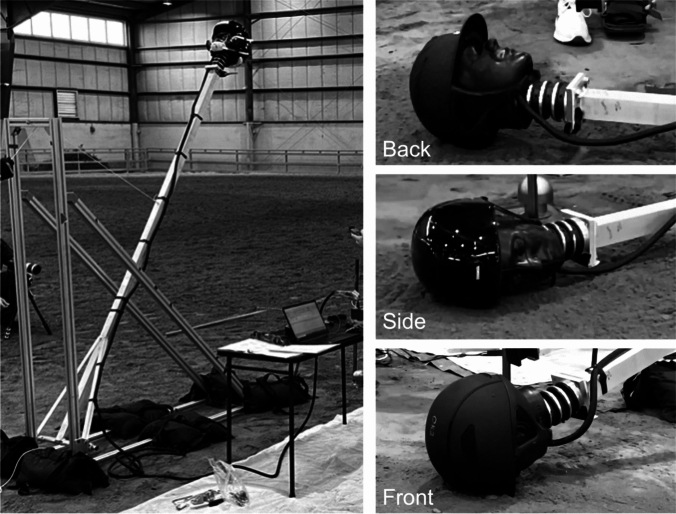
Onsite field-testing setup for a back impact at 6.3 m/s on a dirt surface using the inverted pendulum (**A**) and the three impact locations: back (**B**), side (**C**), and front (**D**)

**Fig. 10:**
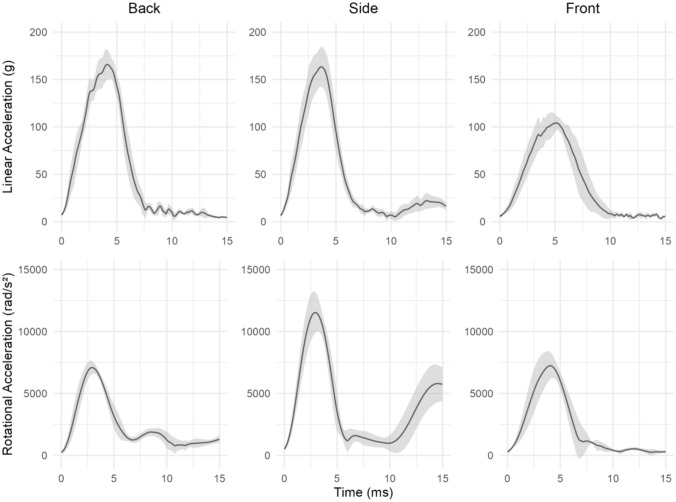
Average linear and rotational acceleration traces for back, side, and front impacts at 5 m/s on the dirt surface. The shading represents one standard deviation.

**Fig. 11:**
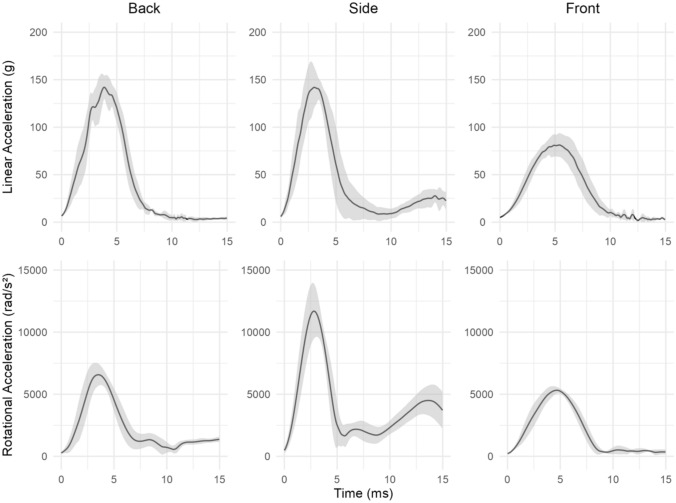
Average linear and rotational acceleration traces for back, side, and front impacts at 5 m/s on the sand surface. The shading represents one standard deviation

**Fig. 12:**
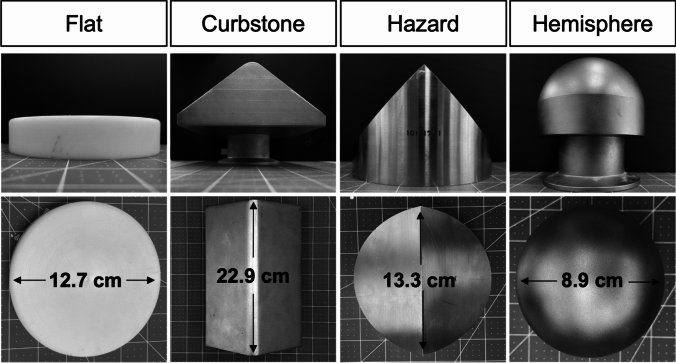
Side and aerial views of the four potential impactor faces

**Fig. 13:**
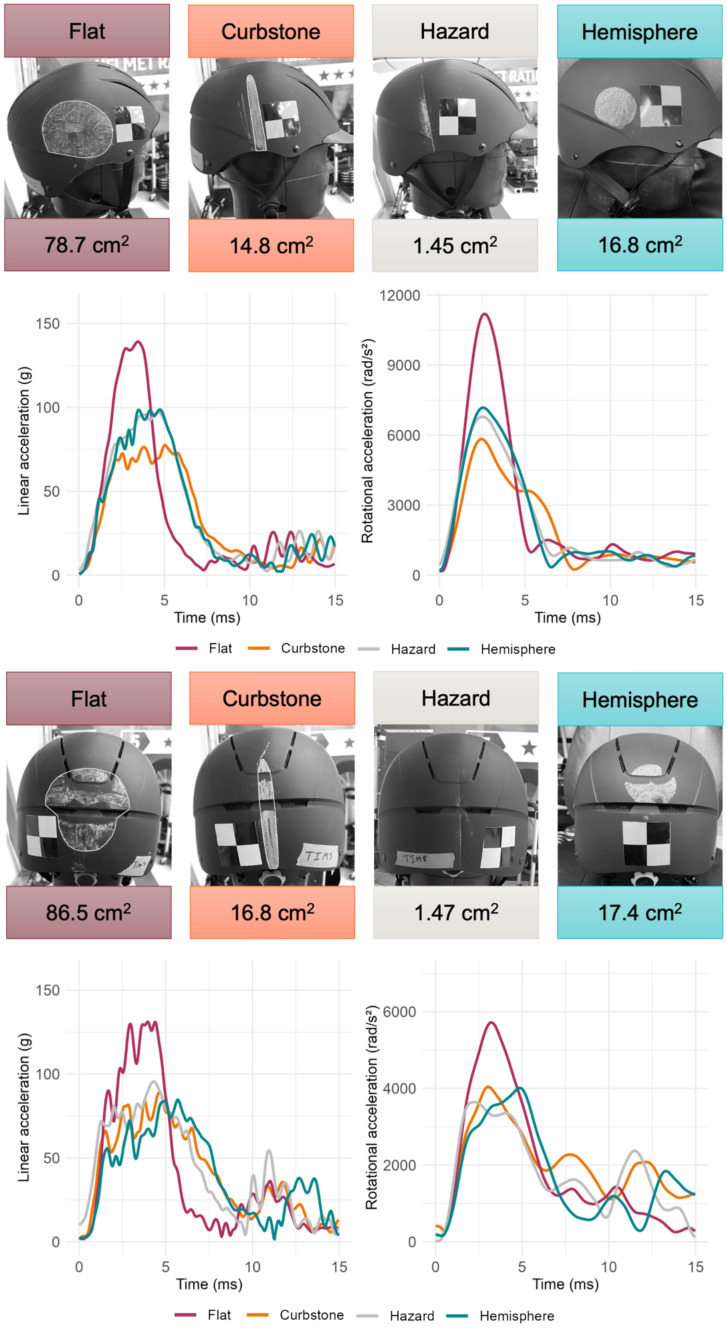
Helmet contact area and linear acceleration and rotational acceleration traces for side impacts (top) and back impacts (bottom) at 5 m/s.

## Video Analysis of Equestrian Falls

To identify the most common head impact mechanisms and locations, 100 videos of real-world equestrian falls were collected and analyzed. Videos were selected from three equestrian events: cross country, show jumping, and dressage. For each fall, an image was taken of the beginning of the fall event, the actual drop height, and the impact location (Fig. 7). The location of each head impact was recorded and designated as front, side, back, or top. Additionally, each head impact was classified as either a high impact or a medium impact. High impacts occurred when the rider fell from the full height of the horse while sitting upright, and medium impacts occurred when the rider grabbed the horse’s neck, reducing the overall fall height.

Each of the 100 recorded falls involved a body impact and 66 of those falls involved a head impact. The most common head impact locations were the back, side, and front of the head. For the 66 head impacts, there were 31 impacts to the back, 24 impacts to the side, and 11 impacts to the front (Fig. 8). There were no impacts to the top of the head. Lower head impact energies were the most common: 73% of the riders that had a head impact fell from a medium reduced height and 27% of the riders fell from the full height of the horse (Fig. 8).

## Onsite Testing of Equestrian Helmets

Onsite testing of equestrian helmets was conducted at the Smithfield Horse Center at Virginia Tech. A portable inverted pendulum was designed and fabricated for the onsite impact tests (Fig. 9). The inverted pendulum was adjustable to different drop heights and impact velocities. A NOCSAE headform was attached to the end of the pendulum arm with a Hybrid III 50^th^ percentile male neck using a custom adaptor plate.

A total of 60 impact tests, with five equestrian helmet models, three impact locations, two impact speeds, and two surface types were performed. The linear and rotational acceleration traces were recorded for each test. Five equestrian helmets ranging in price were selected for onsite testing: the Troxel Sport, Troxel Spirit, Troxel Spirit Low Profile, Ovation Jump Air, and One K Defender. Each helmet model was impacted at three locations, the back, side, and front of the head, at two impact speeds: 5.0 m/s and 6.3 m/s. The back and side impacts were horizontal, and the front impact had 20 degrees y-rotation.

Helmet testing was performed on raked sand and raked and smoothed dirt surfaces. A Clegg impactor (Turf-tech International, Tallahassee Florida) was used to measure the density and impact attenuation of both surfaces. This device uses a 2.25-kg freefalling mass to evaluate the surface stiffness, with firmer surfaces having a higher Clegg Impact Value (CIV). The Clegg impactor is commonly used to evaluate the safety of sporting surfaces, including equestrian surfaces, such as dirt, sand, turf, synthetic, and grass. The sand surface used for testing had a CIV of 30, and the dirt surface had a CIV of 120.

The back, side, and front impacts at 5.0 m/s on the dirt surface had average peak linear accelerations of 165.9 g, 163.4 g, and 104.1 g and average peak rotational accelerations of 7087 rad/s^2^, 11526 rad/s^2^, and 7234 rad/s^2^, respectively (Fig. 10). On the sand surface, the back, side, and front impacts at 5.0 m/s had average peak linear accelerations of 142.0 g, 141.9 g, and 81.3 g and average peak rotational accelerations of 6567 rad/s^2^, 11690 rad/s^2^, and 5322 rad/s^2^ (Fig. 11). On both the dirt and sand surfaces, impacts at 5.0 m/s had impact durations ranging from 6 to 10 ms. Overall, impacts on the dirt surface were more severe with higher peak linear and rotational head accelerations. To evaluate helmets in the most rigorous conditions, helmet testing protocols should focus on head impacts on dirt surfaces compared to sand.

Helmet geometry had an influence on impact severity and duration in the front impact condition. In the front impact tests, the brim of the helmet deformed the dirt or sand surface upon impact. This interaction lowered linear acceleration and increased impact duration in comparison to the back and side impacts, which had little contact between the helmet brim and impact surface.

## Impactor Face Comparison

The potential impactor faces that can be used to test equestrian helmets on a pendulum impactor include the flat, curbstone, hazard, and hemisphere anvils (Fig. 12). The flat anvil is composed of nylon, while the curbstone, hazard, hemisphere anvils are composed of steel. Flat anvils are used across all the current equestrian helmet standards, hazard anvils are used in the ASTM F1163-23, EN 1384:2023, NOCSAE ND050, PAS 015:2011, and SNELL E2021 standards, and the hemisphere anvil is used in the NOCSAE ND050 and SNELL E2021 standards [12, 13, 14, 15, 16].

To determine which impactor face to use for equestrian helmet pendulum testing, the contact surface area and resultant impact response kinematics were measured. For each impactor face, 2 impact tests were performed with the pendulum impactor. Colored chalk was applied to the impactor face, which was then used to impact the back and side of an equestrian helmet at 5.0 m/s. The area of the chalk left on the helmet after each test was recorded along with the linear and rotational acceleration traces.

The flat impactor had the highest peak linear and rotational accelerations for both the side and back impacts (Fig. 13). Sharper impactor faces, such as the hazard anvil, deformed the helmet, reducing peak linear and rotational acceleration. Compared to other impactors, the flat impactor reduced the effect of impactor compliance. The flat impactor also had the highest contact area with the equestrian helmet.

## Helmet Model Performance in Pendulum and Oblique Testing

See Table 7.

**Table 7:** Average peak linear accelerations (PLA) and average peak rotational accelerations (PRA) across pendulum and oblique testing for each helmet model

Brand	Model	Pendulum					Oblique			
		4.0 m/s Average PLA	4.0 m/s Average PRA	6.3 m/s Average PLA	6.3 m/s Average PRA	STAR score component	6.56 m/s Average PLA	6.56 m/s Average PRA	STAR score component	Total STAR score
Champion	Revolve Pro Plus MIPS Jockey	73.6	3576	129.3	6118	1.93	154.1	5442	1.13	3.05
Champion	Revolve Vent-Air MIPS Jockey	84.2	3847	140.8	6862	2.34	157.7	5982	1.41	3.75
Champion	Revolve Vent-Air MIPS Peaked	80.7	3775	139.2	6932	2.28	135.4	4413	0.62	2.90
Champion	Revolve X-Air MIPS	63.8	3356	130.4	6678	1.91	134.5	4848	0.76	2.67
Champion	Revolve X-Air MIPS Peaked	67.0	3629	116.5	6032	1.53	126.4	4703	0.58	2.11
Champion	X-Air Plus Jockey	70.8	3612	127.5	6252	1.72	155.3	7329	1.63	3.36
Charles Owen	4Star	70.2	3956	135.2	6882	2.06	185.3	7653	1.92	3.98
Charles Owen	AYR8 Plus	82.5	3612	146.0	6309	2.18	156.6	7369	1.58	3.76
Charles Owen	EQX Kylo	100.4	4772	158.7	7468	3.89	161.9	5709	1.38	5.27
Charles Owen	Halo MIPS	82.2	4378	147.3	7417	2.91	159.8	5821	1.25	4.16
Charles Owen	MS1 Pro MIPS	91.9	4519	156.7	7873	3.25	152.5	5154	1.08	4.33
Charles Owen	My PS MIPS	92.9	4946	150.6	7997	3.95	159.7	5351	1.28	5.23
GPA	Speed Air 2X	81.9	4034	164.4	7234	2.95	158.6	8451	1.87	4.83
IRH	4G	88.1	3988	178.1	6806	3.24	169.5	8281	1.90	5.14
IRH	Equi-Lite	74.3	3947	153.0	6380	2.50	150.7	6260	1.43	3.92
IRH	Equi-Pro II	96.7	4371	170.2	7333	3.42	172.5	8865	1.94	5.37
IRH	Equi-Pro SV	89.1	4308	168.5	7772	3.21	177.8	8390	1.94	5.15
IRH	Medalist	90.7	4921	186.2	9153	4.39	172.3	7470	1.79	6.18
Kask	Dogma	90.6	4890	162.7	8249	3.91	162.0	7745	1.84	5.74
Kask	Kooki	100.4	5017	168.4	8513	4.79	167.7	8305	1.89	5.10
Kask	Kooki Lady	87.4	4144	149.9	7544	2.84	170.0	8396	1.89	6.68
Kask	Star Lady	92.7	4951	163.8	7791	4.18	160.1	7750	1.80	4.73
KEP	Cromo 2.0	93.8	4596	159.4	8037	3.22	169.9	8144	1.88	5.98
One K	Avance CCS MIPS Wide Brim	97.0	4824	187.9	8513	4.76	168.1	5600	1.42	6.18
One K	Defender	95.9	4778	193.1	7798	4.87	164.0	7774	1.81	6.68
One K	Defender 2023	96.4	4637	171.4	7531	3.93	177.1	9585	1.94	5.86
Ovation	Deluxe Schooler	94.1	4355	179.0	7892	3.81	187.1	9791	1.98	5.79
Ovation	Jump Air	92.9	4565	166.7	7825	3.64	164.1	8082	1.86	5.50
Ovation	Sync	91.5	4268	183.0	7252	3.65	173.6	8664	1.94	5.59
Resistol	Straw Ridesafe	88.5	4675	163.9	9447	3.62	159.3	6018	1.48	5.09
Samshield	Shadowmatt	90.7	3885	175.7	7444	3.31	162.0	7441	1.80	5.11
Tipperary	Sportage hybrid	74.9	3968	149.0	6821	2.39	158.6	8715	1.90	4.30
Tipperary	Windsor MIPS	98.2	4668	172.3	7914	3.81	175.9	6303	1.64	5.46
Trauma Void	EQ3 MIPS	82.8	4072	148.4	7302	2.67	150.7	4938	0.98	3.65
Trauma Void	Lynx Eventing MIPS	83.5	4127	151.9	7151	2.60	146.4	4731	0.82	3.42
Trauma Void	Lynx MIPS	84.0	4244	153.0	7412	2.79	154.3	5565	1.25	4.05
Trauma Void	Pardus MIPS	83.8	4471	156.7	8031	3.30	156.9	4705	1.02	4.31
Troxel	ES	93.0	4400	177.3	6799	3.60	176.6	8744	1.93	5.54
Troxel	Spirit	95.7	4865	147.0	7640	4.36	183.7	8379	1.95	6.31
Troxel	Spirit Low Profile	97.0	4807	177.5	8094	4.32	183.9	7989	1.93	6.24
Troxel	Sport 2.0	98.4	5771	200.0	10499	6.26	168.9	7634	1.78	6.41
Troxel	Terrain	96.1	4828	172.7	8071	4.42	191.1	11182	1.99	8.03
TuffRider	Carbon Fiber	76.5	3763	140.0	7006	2.11	176.9	8075	1.92	4.04
TuffRider	Ventek	86.7	4237	160.0	7208	3.04	155.0	6747	1.59	4.63
Uof	Race	84.4	4383	151.7	7651	2.78	170.7	8962	1.94	4.73

Pendulum testing included front, side, and back impacts at 4.0 m/s and 6.3 m/s, and oblique testing included front boss and rear boss impacts at 6.56 m/s. The STAR scores were calculated using the PLAs and PRAs from these impact tests (Eqs. 1, 2, and 3)
